# Factors associated with pulmonary tuberculosis in elderly individuals: A protocol for a scoping review

**DOI:** 10.1371/journal.pone.0318375

**Published:** 2025-02-13

**Authors:** Francisco de Assis Moura Batista, Juliana Iscarlaty Freire de Araújo, Fernanda Cunha Soares, Thalyta Cristina Mansano Schlosser, Clarissa Terenzi Seixas, Thaiza Teixeira Xavier Nobre

**Affiliations:** 1 Department of Health Sciences, Federal University of Rio Grande do Norte, Natal, Rio Grande do Norte, Brazil; 2 Department of Dental Medicine, Division of Orthodontics and Pediatric Dentistry, Karolinska Institutet, Stockholm, Sweden; 3 Department of Nursing. State University of Campinas, Campinas, São Paulo, Brazil; 4 University Department of Nursing, Paris City University, Paris, França; 5 Trairi Faculty of Health Sciences/Federal University of Rio Grande do Norte, Department of Health Sciences, Natal, Rio Grande do Norte, Brazil; Gulu University, UGANDA

## Abstract

**Background:**

Tuberculosis (TB) is an infectious disease with a significant impact on public health, with the pulmonary form being the most critical. The disease poses numerous risks to the elderly population, as it often manifests concomitantly with other age-related illnesses, thereby complicating its diagnosis and management. Several causal and associated factors contribute to the disease.

**Objective:**

The objective of this manuscript is to present a scoping review protocol aimed at mapping the available literature on factors associated with and contributing to the incidence of Pulmonary Tuberculosis (PTB) in the elderly.

**Methods and analysis:**

The scoping review protocol was developed following the guidelines of the Joanna Briggs Institute (JBI) and the PRISMA-ScR checklist, and it has been registered on the Open Science Framework (DOI: 10.17605/OSF.IO/DHQVP). The databases to be searched include Medline via PubMed, Lilacs, Web of Science, Scopus, Embase, as well as gray literature through Google Scholar and the Brazilian Coordination for the Improvement of Higher Education Personnel (CAPES) Thesis and Dissertation Catalog. The search strategy is grounded on a research question formulated using the PCC acronym (P–Population; C–Concept; C–Context). Peer-reviewed journal articles, scientific books, editorials, conference proceedings, and theses/dissertations published in Portuguese, English, or Spanish between 2014 and 2024 will be included.

**Discussion:**

Elderly individuals are more susceptible to diseases due to the natural decline in immune response to *Mycobacterium tuberculosis*, the causative agent of tuberculosis. In this age group, symptoms are often difficult to detect. Understanding the causal and associated factors of the disease contributes to favorable outcomes and helps reduce the transmission chain to the rest of the population.

## Introduction

Tuberculosis (TB) remains a significant public health challenge worldwide. In 2023, according to the World Health Organization (WHO), an estimated 10.8 million new cases were reported, representing an increase compared to the previous three years. The rise observed between 2021 and 2023 may be linked to disruptions in diagnosis and treatment during the COVID-19 pandemic. This impact on the growing number of cases consequently affects mortality rates from the disease, as well as increases its transmissibility [1].

The disease primarily affects the lungs–Pulmonary Tuberculosis (PTB)–although it can also affect other organs and systems. Pulmonary TB is not only the most common form but also the most significant in terms of public health, especially when sputum smear tests yield positive results, as it plays a central role in the ongoing transmission of the disease [2].

Despite being an ancient illness, TB remains a major public health concern. In Brazil, about 80,000 new cases are recorded each year, resulting in roughly 5,500 deaths due to the disease. Among the elderly population, despite advances in diagnosis and treatment, the incidence rate remains high. In 2023, the incidence rate was reported at a notably high 45.2 cases per 100,000 inhabitants, with 88.9% of these cases manifesting in the pulmonary form [3].

In Brazil, individuals aged 60 and above are classified as elderly [4]. The aging process involves morphological, functional, biochemical, and psychological changes that can increase individual vulnerability, as the capacity to adapt to environmental challenges diminishes with age. The incidence of PTB in the elderly can be attributed to age-related immune decline, the presence of comorbidities, increased sensitivity to medications, and reduced adherence to treatment. Additionally, diagnosing TB in this population can be challenging due to symptom overlap with other prevalent diseases in this age group, such as respiratory, cardiovascular, and systemic conditions [5].

The occurrence of PTB in elderly individuals can be explained by the decline in immunity resulting from the natural aging process, as well as the presence of comorbidities, increased sensitivity to medication, and lower treatment adherence [6,7]. Disease identification in this population may be further complicated by the overlap of symptoms with other conditions prevalent in this age group, such as respiratory, cardiovascular, and systemic diseases [6].

The global rise in life expectancy and the growth of the elderly population underscore the importance of understanding the specific factors influencing PTB management in this age group. Moreover, diagnosing TB in elderly individuals is often delayed due to atypical clinical presentations and symptom overlap with other common diseases in this demographic, such as chronic respiratory diseases and heart failure. This diagnostic delay can worsen the disease’s progression and increase its transmissibility. Therefore, identifying associated factors is essential for disrupting the transmission chain, extending beyond this specific age group.

In light of these considerations, this manuscript aims to present a scoping review protocol intended to map the scientific literature for evidence on factors associated with and contributing to PTB in elderly individuals.

## Methods

This scoping review protocol will be conducted based on the guidelines provided by the Joanna Briggs Institute (JBI) [8] manual for this specific type of study, as well as in adherence to the Preferred Reporting Items for Systematic Reviews and Meta-Analysis Protocols (PRISMA-ScR) extension for scoping reviews [9]. The protocol has been registered on the Open Science Framework (OSF) (DOI: 10.17605/OSF.IO/DHQVP), and any methodological amendments will be updated in the final review version.

To develop the research question, the PCC (Population, Concept, and Context) framework will be applied, where:

- **Population**: Elderly individuals aged 60 years and above;
- **Concept**: Factors associated with and causal to PTB incidence;
- **Context**: PTB diagnosis.

The primary research question guiding this review is as follows: What are the associated and causal factors contributing to PTB in elderly individuals?

### Eligibility criteria

For inclusion in this review, studies must meet the criteria established by the PCC framework that guided the research question (Table 1). Temporal and language restrictions will be applied to document selection, with a focus on cross-sectional and longitudinal observational studies.

**Table 1:** Study eligibility. Natal, RN, Brazil, 2024.

	Inclusion Criteria	Exclusion Criteria
**Population**	Elderly individuals aged 60 years or older, as defined by Brazil’s Elderly Statute.	Studies involving individuals below the defined age threshold.
**Concept**	Risk factors, comorbidities, and social, behavioral, and environmental factors associated with TBP.	Elderly individuals affected by extrapulmonary TB.
**Context**	Cases of Pulmonary Tuberculosis (PTB) in elderly populations, focusing on the clinical and epidemiological context of TB in elderly populations.	Studies involving TB that do not address the review’s research question.
**Sources of Evidence**	The study will consider peer-reviewed journals, textbooks, editorials, conference proceedings, and theses/dissertations available in established databases, in Portuguese, English, or Spanish, from 2014 to 2024.	

Developed by the authors, 2024.

### Information sources and search strategies

The search will include the electronic databases Medline via PubMed, Lilacs via the Virtual Health Library (BVS), Web of Science, Scopus, and Embase. Additionally, gray literature will be searched through Google Scholar and the Catalog of Theses and Dissertations by the Brazilian Coordination for the Improvement of Higher Education Personnel (CAPES).

To identify relevant descriptors, controlled vocabularies were employed: Health Sciences Descriptors (DeCS), Medical Subject Headings (MeSH), and Emtree (for Embase). A preliminary search was conducted to identify free terms for inclusion in the main search strategy. It was observed that, in certain databases, such as PubMed and Web of Science, satisfactory results were achieved using only two terms: "Elderly" AND "Pulmonary Tuberculosis." In these cases, “Risk Factors” will be used as an inclusion criterion. Finally, the search string was adjusted for each database utilized in this review. The specific search strategies for each database are outlined in Table 2.

**Table 2:** Search strategy defined for the scoping review. Natal, RN, Brazil, 2024.

Database	Search Strategy
**PUBMED**	("Tuberculosis, Pulmonary"[mh] OR "Tuberculosis, Pulmonary"[tiab] OR "Consumption*, Pulmonary"[tiab] OR "Pulmonary Consumption*"[tiab] OR "Pulmonary Phthisis"[tiab] OR "Phthises, Pulmonary"[tiab] OR "Phthisis, Pulmonary"[tiab] OR "lung tuberculosis"[tiab] OR "chronic pulmonary tuberculosis"[tiab] OR "chronic tuberculosis, lung"[tiab] OR "lung TB"[tiab] OR "pulmonary TB"[tiab] OR "tuberculous bronchitis"[tiab] OR "lung tuberculosis"[tiab]) AND ("elderly person"[tiab] OR Elder*[tiab] OR "old age"[tiab] OR "older people"[tiab] OR "aged people"[tiab] OR "oldest people" [tiab] OR "older adult*"[tiab] OR senium[tiab] OR "aged people"[tiab] OR "old adult*"[tiab] OR "oldest adult*"[tiab] OR "older person*"[tiab] OR "old person*"[tiab] OR "older patient*"[tiab] OR "oldest patient*"[tiab] OR "geriatric patient"[tiab])
**Web of Science**	("Tuberculosis, Pulmonary" OR "Tuberculosis, Pulmonary" OR "Consumption*, Pulmonary" OR "Pulmonary Consumption*" OR "Pulmonary Phthisis" OR "Phthises, Pulmonary" OR "Phthisis, Pulmonary" OR "lung tuberculosis" OR "chronic pulmonary tuberculosis" OR "chronic tuberculosis, lung" OR "lung TB" OR "pulmonary TB" OR "tuberculous bronchitis" OR "lung tuberculosis") AND ("elderly person" OR Elder* OR "old age" OR "older people" OR "aged people" OR "oldest people" OR "older adult*" OR senium OR "aged people" OR "old adult*" OR "oldest adult*" OR "older person*" OR "old person*" OR "older patient*" OR "oldest patient*" OR "geriatric patient") (Topic)
**SCOPUS**	TITLE-ABS-KEY = ("Tuberculosis, Pulmonary" OR "Tuberculosis, Pulmonary" OR "Consumption*, Pulmonary" OR "Pulmonary Consumption*" OR "Pulmonary Phthisis" OR "Phthises, Pulmonary" OR "Phthisis, Pulmonary" OR "lung tuberculosis" OR "chronic pulmonary tuberculosis" OR "chronic tuberculosis, lung" OR "lung TB" OR "pulmonary TB" OR "tuberculous bronchitis" OR "lung tuberculosis") AND TITLE-ABS-KEY = ("elderly person" OR Elder* OR "old age" OR "older people" OR "aged people" OR "oldest people" OR "older adult*" OR senium OR "aged people" OR "old adult*" OR "oldest adult*" OR "older person*" OR "old person*" OR "older patient*" OR "oldest patient*" OR "geriatric patient") AND TITLE = ("Risk Factor*" OR "Population at Risk" OR "Risk Score*" OR "Risk Factor Score*" OR "Health Correlates" OR "Social Risk Factor*" OR "Factor, Social Risk" OR "Population at Risk" OR risk OR "relative risk" OR "risk hypothesis" OR "Risk Assessment" OR "risk analysis" OR "Analysis, Risk" OR "Assessment, Benefit-Risk" OR "Assessment, Health Risk" OR "Assessment, Risk" OR "Assessment, Risk-Benefit" OR "Assessments, Risk-Benefit" OR "Benefit Risk Assessment")
**EMBASE**	(’lung tuberculosis’/exp OR ’tuberculosis, pulmonary’:ti,ab,kw OR ’consumption*, pulmonary’:ti,ab,kw OR ’pulmonary consumption*’:ti,ab,kw OR ’pulmonary phthisis’:ti,ab,kw OR ’phthises, pulmonary’:ti,ab,kw OR ’phthisis, pulmonary’:ti,ab,kw OR ’lung tuberculosis’:ti,ab,kw OR ’chronic pulmonary tuberculosis’:ti,ab,kw OR ’chronic tuberculosis, lung’:ti,ab,kw OR ’lung tb’:ti,ab,kw OR ’pulmonary tb’:ti,ab,kw OR ’tuberculous bronchitis’:ti,ab,kw OR ’lung tuberculosis’:ti,ab,kw) AND (’elderly person’:ti,ab,kw OR ’elder*’:ti,ab,kw OR ’old age’:ti,ab,kw OR ’older people’:ti,ab,kw OR ’aged people’:ti,ab,kw OR ’oldest people’:ti,ab,kw OR ’older adult*’:ti,ab,kw OR ’senium’:ti,ab,kw OR ’aged people’:ti,ab,kw OR ’old adult*’:ti,ab,kw OR ’oldest adult*’:ti,ab,kw OR ’older person*’:ti,ab,kw OR ’old person*’:ti,ab,kw OR ’older patient*’:ti,ab,kw OR ’oldest patient*’:ti,ab,kw OR ’geriatric patient’:ti,ab,kw) AND (’risk factor’/exp OR ’risk factor*’:ti,ab,kw OR ’population at risk’:ti,ab,kw OR ’risk score*’:ti,ab,kw OR ’risk factor score*’:ti,ab,kw OR ’health correlates’:ti,ab,kw OR ’social risk factor*’:ti,ab,kw OR ’factor, social risk’:ti,ab,kw OR ’population at risk’:ti,ab,kw OR ’risk’/exp OR ’risk’:ti,ab,kw OR ’relative risk’:ti,ab,kw OR ’risk hypothesis’:ti,ab,kw OR ’risk assessment’/exp OR ’risk analysis’:ti,ab,kw OR ’risk assessment’:ti,ab,kw OR ’analysis, risk’:ti,ab,kw OR ’assessment, benefit-risk’:ti,ab,kw OR ’assessment, health risk’:ti,ab,kw OR ’assessment, risk’:ti,ab,kw OR ’assessment, risk-benefit’:ti,ab,kw OR ’assessments, risk-benefit’:ti,ab,kw OR ’benefit risk assessment’:ti,ab,kw)
**LILACS**	(mj:"Tuberculose Pulmonar" OR ti:"Consumpção Pulmonar" OR ti:"Tuberculose do Pulmão" OR tw:"Tísica" OR tw:"Tísica Pulmona" OR tw:"Tuberculosis, Pulmonary" OR tw:"Tuberculosis Pulmonar" tw:"Tuberculosis, Pulmonary" OR tw:"Consumption*, Pulmonary" OR tw:"Pulmonary Consumption*" OR tw:"Pulmonary Phthisis" OR tw:"Phthises, Pulmonary" OR tw:"Phthisis, Pulmonary" OR ti:"lung tuberculosis" OR tw:"chronic pulmonary tuberculosis" OR tw:"chronic tuberculosis, lung" OR tw:"lung TB" OR tw:"pulmonary TB" OR tw:"tuberculous bronchitis" OR tw:"lung tuberculosis") AND (mj:idoso OR ti:idoso* OR ti:anciano OR ti:"pessoa idosa" OR tw:"Pessoas de Idade" OR tw:"elderly person" OR ti:Elder* OR tw:"old age" OR tw:"older people" OR tw:"aged people" OR tw:"oldest people" OR tw:"older adult*" OR tw:senium OR tw:"aged people" OR tw:"old adult*" OR tw:"oldest adult*" OR tw:"older person*" OR ti:"old person*" OR tw:"older patient*" OR tw:"oldest patient*" OR ti:"geriatric patient")
**Google Scholar**	("Tuberculosis, Pulmonary" OR "lung tuberculosis" OR "chronic pulmonary tuberculosis") AND (elderly OR aged OR "old age" OR "aged people" OR "older patient*" OR "old adult")
**CAPES Thesis Catalog**	Idoso AND tuberculose pulmonar

Developed by the authors, 2024.

### Study selection from evidence sources

After extracting data from the databases, the references will be managed using EndNote (Clarivate Analytics, PA, USA) to facilitate reference management and removal of duplicate studies. The selected documents will then be uploaded into Rayyan (Qatar Computing Research Institute, Doha, Qatar), enabling blinded screening by reviewers through Rayyan’s blind mode feature. This step will allow for inclusion and exclusion decisions based on predefined criteria. Two independent reviewers will conduct the selection, and any disagreements unresolved between them will be referred to a third reviewer to reach a consensus.

Prior to data collection, a pilot test will be conducted by all authors to reduce bias and ensure a consistent selection process. Each author will review a sample of documents by title and abstract, screening them according to the established criteria. The team will then discuss any discrepancies and adjust criteria and definitions as needed. Screening will commence only once the team achieves an agreement level of 75% or more, according to Fleiss’ Kappa Statistics [10].

### Data synthesis

The data collected will be analyzed according to the study’s objectives. Data will be organized into tables, and study selection will follow the PRISMA flowchart, adapted for scoping reviews. The primary findings will be highlighted and discussed in relation to other published studies.

The extracted data from Rayyan will be transferred into a matrix created in Microsoft Excel, with independent entries by each reviewer. The data extraction matrix will include two main tables (Tables 3 and 4), as shown below.

**Table 3:** Characterization of selected articles for analysis. Natal, RN, Brazil, 2024.

Article	Title	Author and Year	Journal and Database
Article 1. . .	Article title 1. . .	Author and year of article 1. . .	Journal and database of article 1. . .
Article 2. . .	Article title 2. . .	Author and year of article 2. . .	Journal and database of article 2. . .

Developed by the authors, 2024.

**Table 4:** Studies comprising the sample according to objective, study type, and PTB-associated and causal factors in elderly. Natal, RN, Brazil, 2024.

Article	Objective and Study Type	Associated and Causal Factors of PTB in Elderly
Corresponding article number from Table 3	Objective and study tipe corresponding article number from Table 3	Associated and causal factores of PTB in Enderly corresponding article number from Table 3

Developed by the authors, 2024.

### Preliminary timeline for study phases

Table 5 presents the preliminary timeline for the various phases of the study.

**Table 5:** Stages of the scoping review. Natal, RN, Brazil, 2024.

Stage	Start Date	Completion Date
**Pilot searches to define search terms and refine the strategy**	July 2024	July 2024
**Protocol development**	July 2024	July 2024
**Protocol registration on OSF**	August 2024	August 2024
**Study selection**	August 2024	November 2024
**Data extraction**	November 2024	Not completed
**Data analysis and interpretation**	Not completed	Not completed

Developed by the authors, 2024.

This structured timeline serves as an initial framework to guide the systematic progression of the scoping review. It outlines key activities, ensuring clarity in their execution and facilitating adherence to project milestones.

## Discussion

The elderly population is more susceptible to TB due to reduced cellular immune response to *Mycobacterium tuberculosis*, which increases their vulnerability to both exogenous infection and reactivation of latent bacilli-containing foci. Additionally, symptoms of TB in the elderly are difficult to detect, as they often overlap with clinical manifestations of other respiratory, cardiovascular, and systemic diseases that present similar profiles [11].

TB symptoms in the elderly can be challenging to identify due to the frequent coexistence of respiratory, cardiovascular, and systemic diseases with similar clinical presentations. Moreover, older adults often struggle to report symptoms accurately due to memory deficits, confusion, senility, and verbalization issues, which contributes to diagnostic delays [12].

In the study by Chaves et al. [13], which analyzed 82 medical records of elderly individuals treated at a university hospital between 2009 and 2013, a significant proportion had PTB (75.6%), with nearly half (49.1%) being smokers. The study also found that most elderly patients affected were male, potentially explained by a lower rate of healthcare utilization among men.

Regarding conditions associated with TB, 69.5% of elderly patients reported at least one associated condition, with smoking, alcoholism, diabetes mellitus, hypertension, and AIDS being the most common [12]. In another study by Gloria et al. [14], approximately 89% of the participants had TB associated with at least one additional condition, such as COPD, AIDS, or bronchial asthma.

TB remains a public health challenge, particularly in the elderly population. Identifying factors contributing to the disease in this age group directly supports the development and implementation of public health policies targeting TB in this demographic.

The study on factors associated with PTB in the elderly offers several strengths that highlight its relevance. The research may contribute to a better understanding of the clinical and epidemiological characteristics of this disease in a vulnerable population, often underdiagnosed due to symptom overlap with other common conditions in older adults. Additionally, identifying specific risk factors, such as prevalent comorbidities and risk behaviors, could aid in developing more effective prevention and control strategies tailored to the needs of this age group.

## Conclusion

This protocol has systematized and organized the methodological steps for the scoping review that will be conducted in the subsequent stage following its development. The protocol was constructed in accordance with the guidelines of the Joanna Briggs Institute manual. It is expected that the review, which aims to map the literature on the main associated and causal factors of pulmonary tuberculosis in the elderly, will provide compiled data that contribute to a better understanding of the distribution of cases among older adults and enhance the characterization of the disease in this population. Furthermore, the findings may support the strengthening of more effective public policies aimed at combating the infection within this age group.

## Supporting information

S1 File Prisma-P 2015 checklist. Checklist prism protocol, Natal, RN, Brazil, 2024.
(DOCX)
