# Supplementary material for: Factors associated with pulmonary tuberculosis in elderly individuals: A protocol for a scoping review
Source: PLoS One. 2025 Feb 13;20(2):e0318375. doi: 10.1371/journal.pone.0318375 (PMC11824960; doi:10.1371/journal.pone.0318375)
Supplement: Attachment — Submitted filename: Response to Reviewers.docx [file pone.0318375.s003.docx]

PLOS ONE	December 17, 2024

Editor-in-Chief

Dear Editor:

Attached for your consideration is the revised version of the manuscript entitled "FACTORS ASSOCIATED WITH PULMONARY TUBERCULOSIS IN ELDERLY INDIVIDUALS: a protocol for a scoping review" (PONE-D-24-53195). We are grateful for the time and effort dedicated to reviewing our manuscript. The reviewers' comments were constructive, and we believe they have helped us make significant improvements to the manuscript.

We have made every effort to address the comments and modify the text according to the reviewers' suggestions. The reviewers' comments have been addressed within the revised manuscript (Revised Manuscript with Track Changes).

Should you require any further information, please do not hesitate to contact me.

Sincerely,

Francisco de Assis Moura Batista

(On behalf of all co-authors)
